# Exploring the Linkage between Urban Flood Risk and Spatial Patterns in Small Urbanized Catchments of Beijing, China

**DOI:** 10.3390/ijerph14030239

**Published:** 2017-02-28

**Authors:** Lei Yao, Liding Chen, Wei Wei

**Affiliations:** 1 College of Geography and Environment, Shandong Normal University, Ji’nan 250014, China; alex_yaolei@126.com; 2 State Key Laboratory of Urban and Regional Ecology, Research Center for Eco-Environmental Sciences, Chinese Academy of Sciences, Beijing 100085, China; liding@rcees.ac.cn; 3 University of Chinese Academy of Sciences, Beijing 100049, China

**Keywords:** connectivity, urban flood risk, spatial pattern, imperviousness, rainfall-runoff, rainfall simulation

## Abstract

In the context of global urbanization, urban flood risk in many cities has become a serious environmental issue, threatening the health of residents and the environment. A number of hydrological studies have linked urban flooding issues closely to the spectrum of spatial patterns of urbanization, but relatively little attention has been given to small-scale catchments within the realm of urban systems. This study aims to explore the hydrological effects of small-scaled urbanized catchments assigned with various landscape patterns. Twelve typical residential catchments in Beijing were selected as the study areas. Total Impervious Area (*TIA*), Directly Connected Impervious Area (*DCIA*), and a drainage index were used as the catchment spatial metrics. Three scenarios were designed as different spatial arrangement of catchment imperviousness. Runoff variables including total and peak runoff depth (*Q_t_* and *Q_p_*) were simulated by using Strom Water Management Model (SWMM). The relationship between catchment spatial patterns and runoff variables were determined, and the results demonstrated that, spatial patterns have inherent influences on flood risks in small urbanized catchments. Specifically: (1) imperviousness acts as an effective indicator in affecting both *Q_t_* and *Q_p_*; (2) reducing the number of rainwater inlets appropriately will benefit the catchment peak flow mitigation; (3) different spatial concentrations of impervious surfaces have inherent influences on *Q_p_*. These findings provide insights into the role of urban spatial patterns in driving rainfall-runoff processes in small urbanized catchments, which is essential for urban planning and flood management.

## 1. Introduction

Rapid urban sprawl brings significant landscape modifications, of which the most pervasive hallmark is considered to be the transformation from natural lands to imperviousness [1,2]. This alteration leads to negative hydrologic impacts that result in enhanced hydraulic efficiency and can thus increase stormwater runoff volumes, flow rates and peak flows and flow-time reductions in urban catchments [3,4]. These unwanted side effects of rapid urbanization can increase the susceptibility towards urban flooding which endangers life, private property and public infrastructures and poses substantial threats to urban environmental development [5]. Furthermore, urban flooding risk will possibly intensify as a result of inadequate urban landscape planning and weather extremes, particularly in the context of global climate change [6,7]. Consequently, urban areas are increasingly losing the capacity to cope with the complicated hydrological alterations, and this complexity poses significant challenges for urban flood management. 

How to mitigate excess runoff risks in urban areas is an important question to address, which thus requires a better understanding of hydrological alterations and their linkages to the spatial patterns of urbanization [8,9]. Imperviousness has been recommended as an effective spatial metric to indicate the urbanization level and reflect hydrological alternation [4,10]. For example, Schueler et al. [11] proposed an impervious cover model to diagnose the severity of future urban hydrological problems. Quantitative analysis also was conducted to determine the relationship between imperviousness and total runoff depth [12,13]. However, the significance of imperviousness in predicting other important runoff variables (e.g., peak runoff), has been estimated to be lower than that of total runoff depth, and varied with different rainfall conditions [14], indicating other spatial factors exert influence on urban runoff. A number of studies have stressed that the spatial arrangement of the impervious area and drainage network structures (e.g., drainage density, width function) have additional influence on surface hydrology, particularly on peak flow [8,9,15]. Most of the studies on this focus were conducted in large urban basins (>1 km^2^, with natural stream channels) rather than the realm of the urban system [7]. However, those small-scaled catchments, with the similar size of neighborhood (several hectare), flat terrain, closed hydrological boundary and discharge overland flows mainly through artificial drainage networks [16], were facing increasingly pluvial and overland floods due to drainage blockage [17,18] and unreasonable landscape planning [19,20]. Potential downscaling from large basin to small urban catchment may intensify or weaken the hydrological effects of spatial patterns [21,22]. Due to such complexity, it is still unclear regarding the roles of spatial patterns in runoff responses at small urban catchments. Therefore, it is necessary to reconsider the principles linking hydrological processes to spatial patterns for small urban catchments.

Hydrological models have advantages to assess the impacts of spatial patterns on urban rainfall-runoff processes at multiple spatial scales. Geophysical factors that affect catchment runoff, such as soil condition, climate and land cover, can be easily configured by altering the input parameters in hydrological models [21,23]. Furthermore, hydrological models provide a reductionist way of characterizing various spatial scenarios in detail without considering the complexity of the real world [24,25,26]. As such, model-based analysis is necessary to generalize the specific hydrological process, and thus provides guidance for future urban storm water management efforts [27].

In this study, several typical residential sites with hypothesized drainage systems were selected as the study catchments. The spatial characteristics of these catchments, including imperviousness and land cover types, were identified based on remote sensing methods. Moreover, three different scenarios with different spatial arrangements of the impervious areas were developed. A semi-distributed hydrological model was used to simulate the catchment hydrological responses (i.e., total and peak runoff). The detailed objectives of this paper are posed as follows:

(1) By using regression analysis, this study attempts to quantify the potential relationships between spatial characteristics and urban flood variables under different rainfall conditions for small urbanized catchments;
(2) Comparing the catchment runoff outputs of different scenarios and investigating whether different catchment patterns affect runoff process; and
(3) We discussed the potential implications of our findings and the relevance of the study to urban catchment design and flood management.

## 2. Materials and Methods

### 2.1. Study Areas

**Figure 1:**
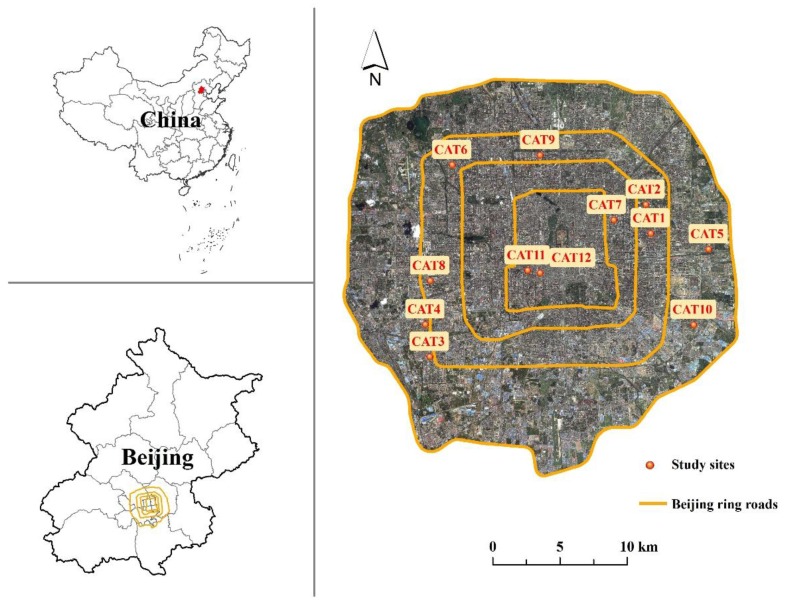
Locations of the selected residential sites in Beijing.

**Figure 2:**
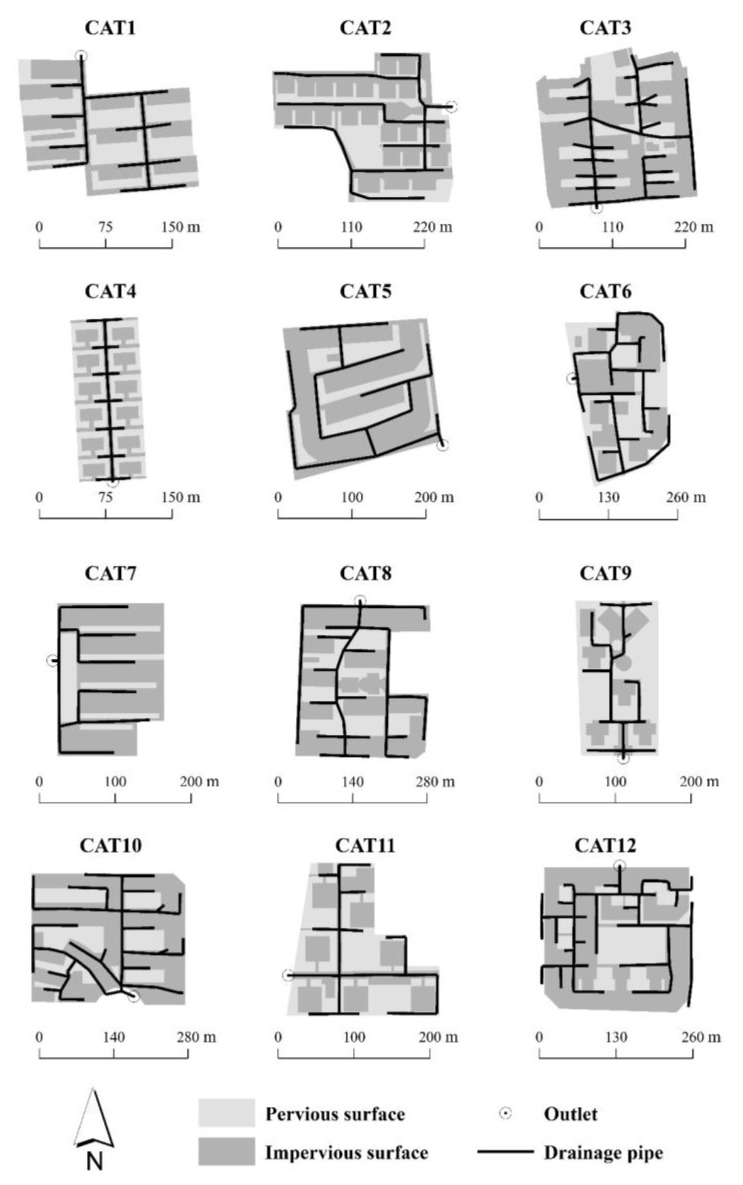
Impervious and drainage layouts of the twelve residential catchments.

The residential sites selected for study are located in Beijing, China (Figure 1). These twelve residential sites, ranging from 1.39 to 6.84 ha in area, show typical building layouts (including linear, interspersed, and semi-enclosed building forms), and are composed of both impervious surfaces (roofs and roads) and pervious surfaces (trees and lawns). Figure 2 presents an overview of the twelve residential sites. Table 1 lists the values of different landscape variables for each of the twelve residential sites. Land cover and Total Impervious Area (*TIA*) of each site were obtained by visual interpretation based on an IKONOS image with 1-m spatial resolution (Table 1).

**Figure 3:**
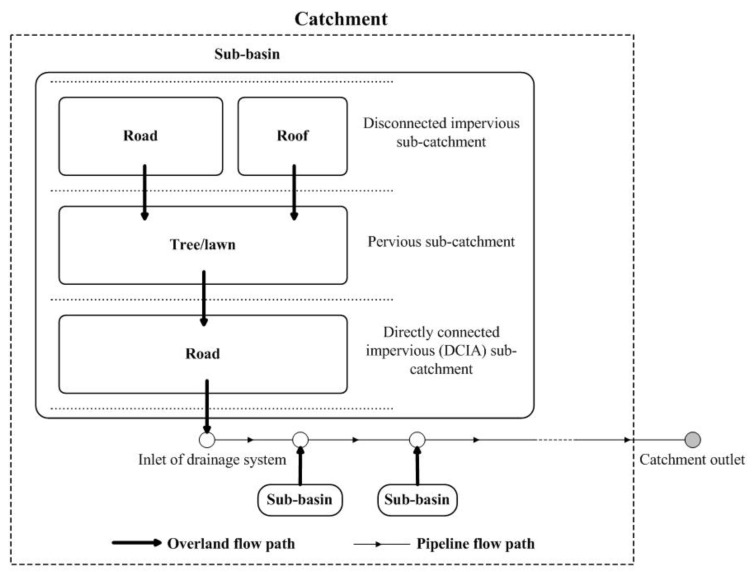
Illustration of catchment structure and flow pathways in SWMM.

Each residential site is considered as one drainage catchment and occupies an independent drainage system. Meierdiercks et al. [8] and Ogden et al. [15] found that different drainage densities play an important role on discharge alterations in urbanized catchments. In order to emphasize the hydrological role of catchment spatial patterns, drainage densities of the twelve residential catchments were hypothesized with the similar drainage density. Catchment drainage pipelines were outlined mainly along the roads according to Goldshleger et al. [28], as shown in Figure 2. The drainage densities of the twelve catchments range from 296.15 to 316.86 m/ha (Table 1). Drainage network morphologies were simplified to share the same values, including the pipeline geometry (circle, 1-m in diameter), minimum slope (2%), and maximum water depth of inlet (2-m). In addition, following the method presented by Lee and Heaney [29] and Yao et al. [30], the Directly Connected Impervious Area (*DCIA*) of each catchment was determined as the part of impervious surfaces (including roads and a portion of the roofs) which drained the overland flows directly into the drainage networks (Figure 3).

### 2.2. Model Implementation

Complex urban morphology and ungauged conditions make it intrinsically difficult to obtain rainfall-runoff data and to assess the consequences of urbanization [27,31]. In this study, the drainage networks of the catchments were assumed and no monitoring rainfall-runoff data were available, a hydrological model-based analysis thus was conducted to accomplish our goals. All the rainfall-runoff processes for this study were simulated using the Strom Water Management Model (SWMM) [27,31]. SWMM is a semi-distributed hydrological model which treats urban catchments as the “Catchment-Sub-basin-Sub-catchment” structure, as shown in Figure 3. Each sub-catchment is characterized by single land cover type and homogenous properties. As shown in Figure 3, all the sub-catchments can be categorized into three types to identify the detailed flow path and spatial hierarchies: disconnected impervious sub-catchments, pervious sub-catchments, and DCIA sub-catchments [30,32]. The three types of sub-catchments assigned with the same drainage inlet constitute a relatively independent drainage sub-basin. All of the sub-catchments within each sub-basin are connected via the storm runoff flow pathways associated the drainage network in an urban catchment, including overland flow and flow through the drainage pipes. Rainfall-runoff process of each sub-catchment is calculated according to its hydrologic characteristics, such as depression and infiltration losses. Generated overland flows are routed over the sub-catchments to their outlet (i.e., other sub-catchment or drainage network) by using the nonlinear reservoir equation, a combination of the continuity and Manning’s equation [33]. 

**Figure 4:**
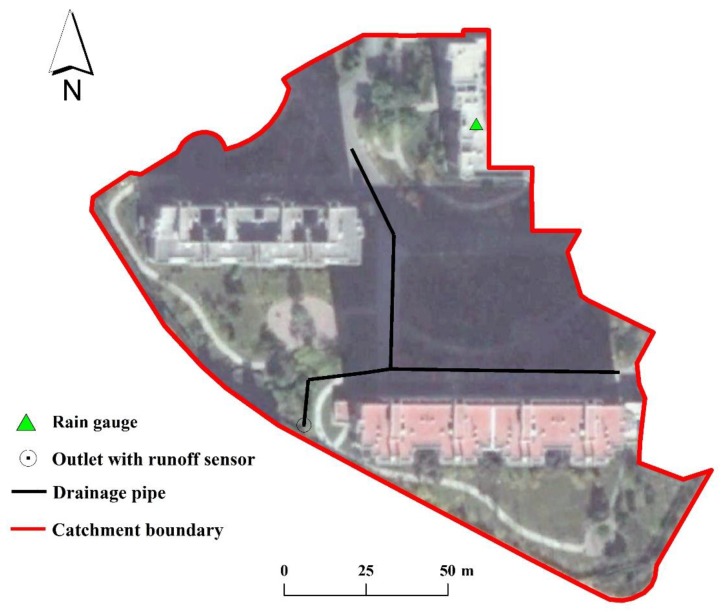
Catchment for rainfall-runoff monitoring in this study.

Twelve models thus were built for the study catchments, and the three types of sub-catchment for each model were delineated based on the catchment land cover and impervious characteristics. In these models, infiltration losses of pervious surfaces were estimated using Horton equation, and the Kinematic Wave method was used for runoff routing computation for drainage networks [34]. Determination of model parameters are described as follows:

(1) Spatial parameters in SWMM model including drainage area, flow width [32], impervious coverage, and drainage pipeline length were calculated according to the geo-analysis of the GIS data (Figure 2).
(2) As previously described, drainage systems of the study catchments were designated to be similar in order to minimize the hydraulics interference from drainage structures. Therefore, no real life measurements were conducted in these sites, and no suitable rainfall-runoff datasets are available for model calibration/validation. To guarantee the model credibility, we conducted a hydrological monitoring task in a residential catchment of Beijing, China (40°2′ N, 116°24′ E), as shown in Figure 4. This monitored catchment covers a drainage area of 1.69 ha. We collected detailed rainfall-runoff data in this site with runoff sensor and rain gauge during the rainy season in 2013. Following the modelling procedure mentioned above, we built a detailed model for this monitored catchment by using SWMM model. Then we obtained a group of calibrated SWMM parameters based on these rainfall-runoff data (Table 2). More details about this monitored catchment and its model calibration/validation process can refer to Yao et al. [14].

After side-walk investigations, we confirmed that this monitored catchment shared the similar land cover with our study catchments, i.e., roofs, roads, trees, and lawns. This study thus adopted these calibrated parameters in Table 2 for all these built models equally. This reductionist parameterization approach can guarantee the same hydrological conditions among these study catchments and minimize the significant hydrological disturbances from diverse model parameters. However, this of course introduces a certain level of uncertainty to the results presented in this work. To handle this uncertainty, it is necessary to interpret the simulated model results in an appropriate way. Following the similar purport presented by Vos et al. [35], this study mainly focus on explaining the general hydrological trends that show up from the multitude of study catchments rather than individual results. In this way, we believe that our modeling results do have a valuable role and thus guarantee a fair quantitative characterization on the hydrological effect of spatial patterns in small urbanized catchments. 

### 2.3. Input Rainfall Conditions 

**Figure 5:**
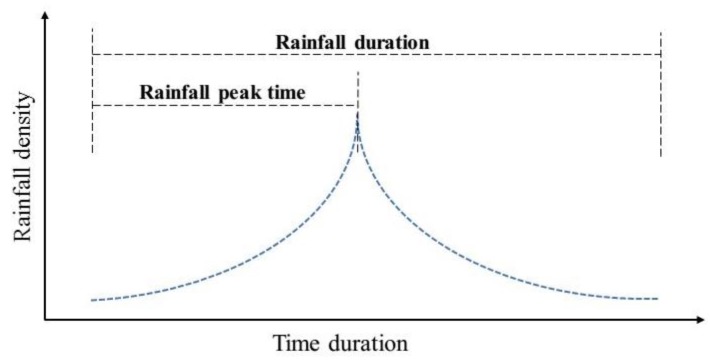
Designated rainfall hyetograph by using the Chicago hyetograph method.

A group of rainfall events with various return periods were designated as the SWMM input data based on the rainfall intensity formula in Beijing and the Chicago hyetograph [26,36]. Eventually, rainfall events with the return periods of 0.1, 0.15, 0.2, 0.3, 0.5, 1, 3, 5, and 10 year, which had the same rainfall duration (2-h) and peak ratio (0.4, the ratio of rainfall peak time to rainfall duration, as shown in Figure 5) were adopted in this study. The corresponding total rainfall amounts were 8.8, 10.9, 20.2, 26.9, 35.3, 46.6, 64.7, 73.1, and 84.5 mm, respectively. A wide range of rainfall amounts can encompass both the minor and major storm conditions. All the rainfall data were input to the built SWMM models separately, and the simulated runoff results could be obtained from the output files of the models.

### 2.4. Data Analysis 

**Figure 6:**
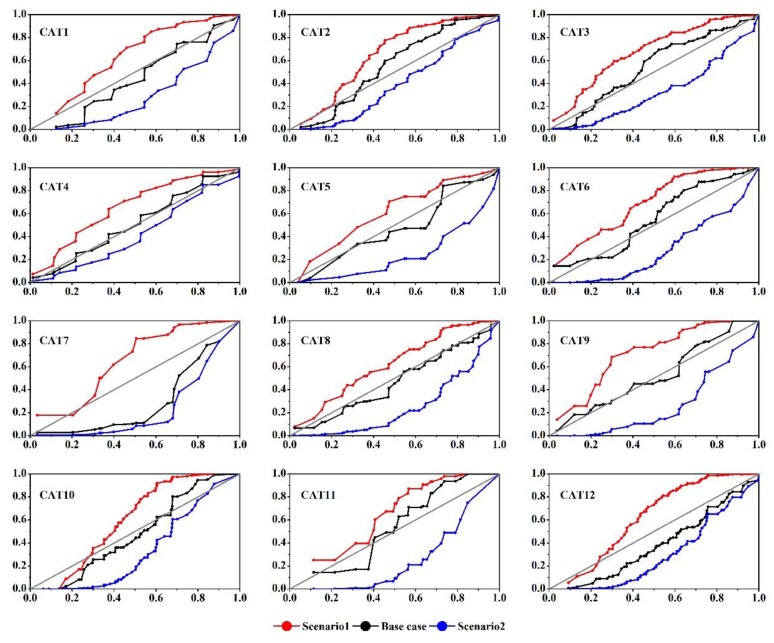
Impervious Area Curves (*IAC*) of the twelve catchments for different scenarios. *X*-axis represents the fraction of flow distance to the catchment outlet; *Y*-axis represents the accumulate fraction of the Total Impervious Area (*TIA*). Grey solid lines represent the uniform distribution of *TIA* in ideal state.

In this study, spatial patterns of the twelve catchments were characterized by using *TIA*, *DCIA*, average drainage area (*A_d_*), and Impervious Area Curves (*IAC*). *TIA* can characterize the overall urbanization level of the catchment, which is significantly related to the catchment runoff generation [13]. *DCIA* is an important attribute of *TIA* that is directly connected to the drainage systems. This metric can represent the hydrological efficiency of impervious surfaces and usually contribute most of the runoff to the whole urban catchments [29,37]. *A_d_* can be treated as an effective index representing both the characteristics of catchment spatial segmentation and drainage structure related to the catchment size and drainage junctions. Higher *A_d_* represents larger drainage area and drainage loads assigned with each rainwater inlet, which may exert potential influence on the catchment hydrograph. *IAC* depicts the accumulated *TIA* fraction as a function of flow distance from the catchment outlet, as presented by Meierdiercks et al. [8]. This type of curve can reflect the spatial configuration of catchment impervious surfaces along with the drainage pathway. Specifically, accumulated *TIA* faction was presented the curves, and the corresponding flow distance from the outlet was shown on the *X*-axis, as shown in Figure 6. 

Runoff volumes and peaks are commonly used to study the impacts from urbanization on watersheds [9,38]. Two key indicators, total runoff depth (*Q_t_*, mm) and peak runoff depth (*Q_p_*, mm/min), were used to represent the catchment runoff responses. *Q_t_* and *Q_p_* of each catchment under all the designated rainfall conditions were calculated from the model output files, and the detailed calculation procedures for these two indicators were presented by Yao et al. [14]. Basic description statistics was used to describe the details of spatial characteristics and runoff responses of the catchments. Multiple linear regression models were developed to describe the significance of spatial patterns in runoff variables assigned with various rainfall conditions. Three scenarios were designated to examine the potential hydrological alterations with different impervious locations. We rearranged all the sub-basins with different rainwater inlets (Figure 3) of each catchment in the SWMM model, in order to obtain different impervious concentration scenarios without changing impervious fraction. Different impervious location scenarios could be depicted with various *IAC*s. The original catchment *IAC* was for the base scenario. Two additional scenarios were determined to downstream and upstream concentration of imperviousness, respectively, as shown in Figure 6.

## 3. Results

### 3.1. Spatial Details of the Study Catchments

As shown in Table 1, *A_d_* of the twelve catchments ranged between 0.07 and 0.18 ha. The catchment fractions of *TIA* and *DCIA* showed large variations, ranging from 37.95% to 77.79% and 19.27% to 71.42%, respectively. Roof surfaces contributed most of the *TIA* for all the catchments except CAT 9, whereas road surfaces contributed the major *DCIA*. The general distribution characteristics of impervious surfaces for the twelve catchments also showed great differences based on the gaps between *TIA* and *DCIA*. Among the catchments, CAT 4 and 11 had the greatest difference between *TIA* and *DCIA*, with 30.32% and 29.29%, respectively; the least gap was found in CAT 10 and only 3.08% of the land surfaces were treated as disconnected impervious area, which equals to the part of *TIA* excluding *DCIA*.

### 3.2. Simulated Runoff Variables

At the base case, the runoff responses among the catchments varied significantly. For example, under rainfall with 0.1 year return period, the catchment *Q_t_* ranged from 1.59 to 7.46 mm and *Q_p_* ranged from 0.04 to 0.16 mm/min. In addition, the changing range of *Q_t_* and *Q_p_* of each catchment varied as the rainfall amount increased from 8.8 to 84.5 mm, such as CAT 4 (*Q_t_*: 1.84–67.71 mm) and CAT 6 (*Q_t_*: 3.04–64.75 mm). Detailed simulated runoff data are shown in Table 3. 

### 3.3. Relationship between Catchment Spatial Patterns and Runoff Responses

Multiple linear regression analysis results are shown in Table 4. The results showed that the spatial pattern indicators could predict the runoff responses well (*R^2^* > 0.75) under various rainfall conditions. Particularly, *Q_t_* could be predicted solely by impervious metrics, i.e., *TIA* and *DCIA*, whereas A_d_ acted as a significant indicator (negative) for predicting *Q_p_* besides *TIA* and *DICA*. 

However, the relative significances of *TIA* and *DCIA* in predicting runoff variables varied in different rainfall conditions. *DCIA* generally played a more important role in affecting both *Q_t_* and *Q_p_* than *TIA* when the rainfall return period less than 0.3 year, while *TIA* acted a more dominant role in affecting the two runoff variables than *DCIA* for larger rainfall events. In addition, the rapid decreased coefficient of *A_d_* (from −0.511 to −9.719) indicated that the role of the drainage area in explaining the variations in *Q_p_* became more important as rainfall increased. 

Scenario design significantly altered the original *IAC*s, as illustrated with red and blue lines in Figure 6. *IAC*s in scenarios 1 and 2 depicted curves above and below the grey line for all the catchments, showing obvious impervious concentration down- and up-streams. 

**Figure 7:**
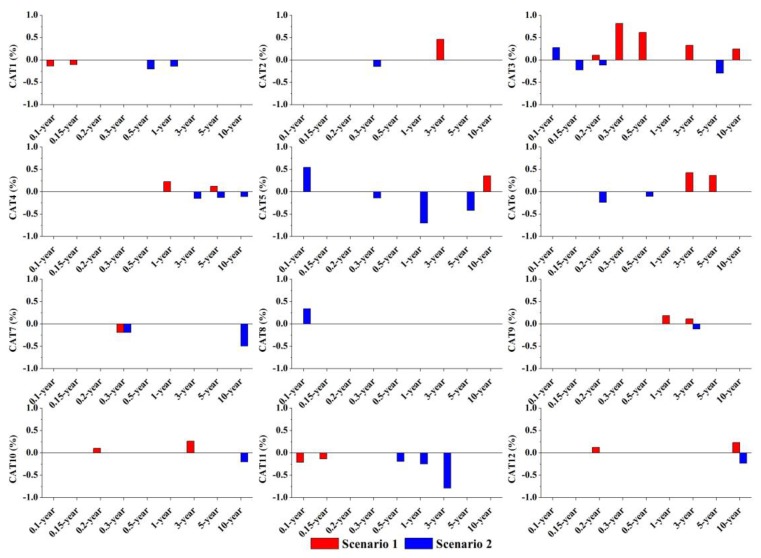
Variations in catchment total runoff depth (*Q_t_*) under different scenarios and rainfall conditions. *X*-axis represents different rainfall conditions assigned with return period; *Y*-axis represents the change rate in *Q_t_* (%) under the scenarios 1 and 2 compared with that in the base case.

Detailed runoff variations of *Q_t_* and *Q_p_* in scenarios 1 and 2 are shown in Figure 6 and Figure 7. Compared to the base case, most of the catchments showed intuitively similar changing trends in both *Q_t_* and *Q_p_* that increasing in scenario 1 and decreasing in scenario 2. However, the changed IACs induced different alterations in *Q_t_* and *Q_p_*. For *Q_t_*, catchments did not experience apparent changes in Q_t_ under different IAC scenarios and rainfall conditions, with the highest variation in *Q_t_* < 1%. Changes in *Q_p_* were more significant than that of *Q_t_*. The highest increment in *Q_p_* was found in CAT under scenario 1, with nearly 4%, while the largest decrement was in CAT 6 under scenario 2 (−8.8%). However, *Q_p_* in most of the catchments experienced a relatively low variation <5%.

## 4. Discussion

### 4.1. Hydrological Impacts of Catchment Spatial Characteristics

The regression results showed that impervious metrics acted as effective indicators in predicting runoff processes in urban catchments. However, the relationship between imperviousness and runoff in urban catchments varied for different rainfall magnitudes (Table 4). The rainfall threshold can be identified between 20.2 and 26.9 mm for both *Q_t_* and *Q_p_*. As rainfall was less than this threshold, *DCIA* performed as one of the most important contributors for runoff; on the contrary, runoff variables were more relevant to *TIA*. This is mainly caused by changing runoff contributions of pervious surfaces under different rainfall magnitudes [12,39]. If rainfall is too small to fully meet the needs of the infiltration losses of pervious surfaces, the catchment runoff is mainly contributed by *DCIA*, whereas part of the overland runoff flow from disconnected impervious surfaces may be infiltrated into the pervious surfaces. As rainfall increases, increased runoff from pervious and disconnected impervious surfaces will attenuate the contribution of *DCIA* for the catchment runoff. This explains why the regression coefficient of *DCIA* decreased as rainfall return period increased (Table 4). After rainfall exceeds the threshold, pervious surfaces tend to saturate and generate steady pervious runoff, so that most of the overland flow from *TIA* can be transferred to the drainage system. The catchment runoff variables including *Q_t_* and *Q_p_* thus are mainly contributed by *TIA*. 

However, this rainfall threshold varies among different studies. Boyd et al. [12] reported that pervious runoff occurred after the rainfall depth was >10 mm in the urban catchments of Australia; while Sillanpää and Koivusalo [40] reported a rainfall threshold of 17–20 mm in a developing residential catchment. The variation in rainfall threshold is close related to the soil conditions of the study catchments, such as antecedent soil moisture and soil type [12]. Guan et al. [41] thus tested the potential influence of different soil infiltration parameters and obtained a threshold greater than 35 mm with high soil infiltration parameters in an urbanized catchment. 

The results also showed that *A_d_* was significantly related (negatively) to the catchment *Q_p_* besides imperviousness. This indicates that changing catchment segmentation condition by altering the number of drainage inlets has a certain influence on *Q_p_* in small urbanized catchments. Namely, the larger the average sub-basin size, the lower peak runoff generated out of the catchment. Sheeder et al. [42] found a positive linear relationship between runoff lag time and catchment size by developing a regression equation. Berne et al. [43] and Di Lazzaro [44] also reported an increasing trend of runoff lag time with larger drainage area via monitored catchment data. As the time required for a given amount of rainfall to runoff shortens, the runoff peak thus will increase, which can explain the negative relationship between *A_d_* and *Q_p_*. 

### 4.2. Effects of Different Catchment Impervious Patterns on Rainfall-Runoff

**Figure 8:**
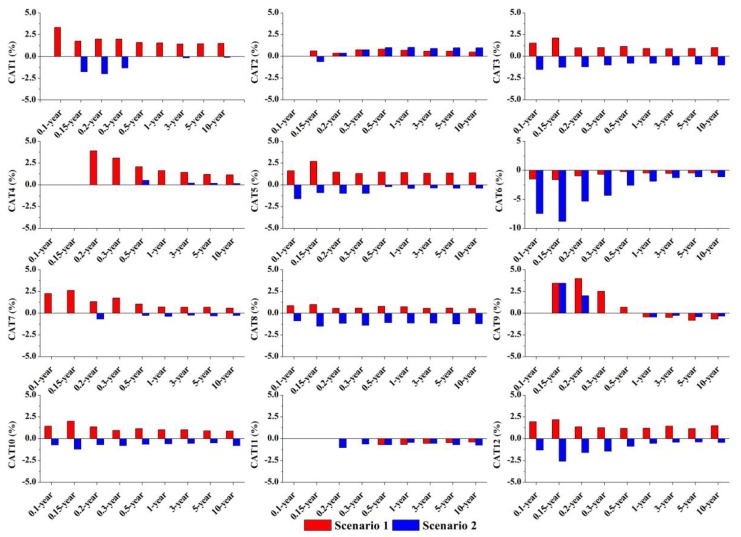
Variations in catchment peak runoff depth (*Q_p_*, %) under different scenarios and rainfall conditions. *X*-axis represents different rainfall conditions assigned with return period; *Y*-axis represents the change rate in *Q_p_* (%) under the scenarios 1 and 2 compared with that in the base case. Specifically, change rate in CAT6 shows different *Y*-axis scale with other catchments.

With different *IAC* scenarios, Figure 7 shows that *Q_t_* basically had no changes (<1%) because it is closely related with the net discharge of different land surfaces [33]. In this study, *IAC* scenario did not alter the size of each type of land surface but only change the spatial arrangement of *TIA* in the catchment. More obvious variations in *Q_p_* indicates that changing *TIA* distribution does have a certain influence on catchment peak discharge (Figure 8). Specifically, the catchments considered in this study generally have higher peak runoff if impervious surfaces concentrate near the catchment outlet, while lower *Q_p_* will be generated with a large fraction of impervious surfaces in the upstream section of the catchment. This difference may be a result of various runoff travel times caused by different impervious distribution [21]. With a higher impervious concentration near the outlet, a catchment tends to produce a faster runoff response and higher peak flows [45]. On the contrary, upstream concentrated impervious conditions may extend the overall catchment runoff travel time and thus restrains the catchment peak discharge volume. 

However, the variation trends of *Q_p_* in the two *IAC* scenarios are not strict. In scenario 2, CAT 4 experienced a slight increment in *Q_p_*, with an average value of 0.12% for all rainfall conditions (Figure 8). These unexpected changing trends in *Q_p_* may be caused by the variation of the runoff hydrograph between that from impervious areas and the whole catchment. More apparent differences between the two types of runoff hydrograph will alleviate the catchment peak runoff discharge [21,46]. Besides the spatial arrangement of imperviousness, other potential factors such as sub-catchment size and shape [45,47], land cover configuration [20,42], and drainage width function [48] could have a certain influence on the runoff processes. These complexities may lead to an intensification or offset *Q_p_* with the changed *IAC* in the catchments considered by this study.

### 4.3. Practical Implications of Results

Controlling total and peak runoff volumes is still the most important goal for urban stormwater management [18,38,49]. This study illuminates the significant influences on *Q_t_* and *Q_p_* of spatial patterns in small urbanized catchments and thus provides specific guidelines for urban landscape design in order to solve urban flood issues. The results suggest that impervious restriction is still the primary measurement for mitigating urban runoff risk. This is supported by Palla and Gnecco [1] who found that the runoff hydrograph tends to come closer to the pre-development condition by reduced the urban catchment imperviousness. However, the two types of impervious metrics (*TIA* and *DCIA*) should distinguish to treat according to the specific rainfall condition, i.e., *DCIA* should be treated as the major contributor to the catchment hydrological disturbance during minor rainfall conditions while *TIA* becomes more important as the rainfall amount increased. Similar findings were documented by Sillanpää and Koivusalo [40] and Guan et al. [41]. In addition, the significant role of *A_d_* shown in Table 4 implies that optimizing sub-basin drainage size by adjusting the number of rainwater inlets can bring a similar effect in mitigating *Q_p_* to that by limiting imperviousness. This is similar to the strategy of low impact development (LID) that keeps the overland flows on site as long as possible instead of draining them away directly [50]. But larger sub-basin areas without adequate stormwater treatment facilities tends to generate higher overland flow volumes (*Q_t_* × *A_d_*) which will increase the drainage pressure of each rainwater inlet and induce sewer overflows and waterlogging risks [18,51]. This latent confliction needs to be balanced with a proper drainage inlets design scheme. Furthermore, a reasonable arrangement of catchment IAC can mitigate the catchment runoff peaks. Since land in urban region is valuable due to development pressure, limiting impervious area is often politically unfeasible due to conflicting economic interests [52,53]. Therefore, this kind of landscape rearrangement performs as a reasonable and cost-effective strategy to relieve urban flood risk without the need for additional cuts in construction land area. It suggests a forethought for the optimization of the architectural arrangement is needed for urban design [47,49]. 

In this study, however, the regulation potential is limited (<5%, except that of CAT 6 < 9%) and dampens as rainfall increases (Figure 8). This is consistent with the results in Table 4, relatively high *R*^2^ (>0.79) of the regression models indicates that those static spatial metrics, i.e., *TIA*, *DCIA*, and *A_d_*, are the most important factors in driving *Q_t_* and *Q_p_* in small urban catchments. Smaller spatial scale with more sensitive runoff responses may restrain the effect of *IAC* on rainfall-runoff. Previous studies found a 30% change in peak flow by altering the *TIA* distribution in a sub-urban basin (54 km^2^), USA [54]. By contrast, Meierdiercks et al. [8] found only a 9% decrease in peak runoff and a 2 min delay in lag time by altering the *TIA* distribution in a small urbanized catchment (1.92 km^2^). Therefore, the hydrological benefit through urban spatial pattern optimization in small urban catchments may prove more productive at larger urban regions, such as the city block or district.

This study assumed the 12 residential catchments with the similar drainage system density (Table 4). Actually, besides imperviousness, drainage efficiency has been widely reported to have significant influences in urban runoff [55,56]. It was reported that the efficiency of the drainage network control is more effective in reducing peak runoff than reducing the effective imperviousness [8,57]. Therefore, a more comprehensive consideration is needed for urban stormwater management with proper impervious configuration and reasonable drainage system arrangement in future urban design. 

## 5. Conclusions

This study used a hydrological model to link the rainfall-runoff processes to spatial patterns in 12 small residential catchments (1.39–6.84 ha). The drainage density of these catchments was hypothesized to be similar (around 300 m/ha). Impervious metrics and average sub-basin drainage size were used to describe the catchment spatial patterns. In addition, we designed three *IAC* scenarios to examine the potential influence of the position of impervious areas on catchment runoff. All these tasks were investigated under different rainfall conditions. Here are the main conclusions:

(1) Imperviousness metrics act as effective indicators in predicting both *Q_t_* and *Q_p_*. However, this significant relationship is rainfall-dependent. For small rainfall conditions, *DCIA* contributes to the majority of hydrological alternations in *Q_t_* and *Q_p_*, while *TIA* becomes more responsible in predicting runoff responses than *DCIA* as rainfall increases.
(2) Sub-basin segmentation of the catchment (*A_d_*) also has essential impacts on its peak runoff. Reasonably reducing the drainage inlet number will benefit peak flow mitigation at the catchment outlet.
(3) Changing the spatial arrangement of imperviousness (*IAC*) could affect the catchment peak runoff. Generally, catchments with an impervious concentration far from the outlet tend to produce a lower peak runoff than that with downstream concentration. However, the regulation potential of *Q_p_* through *IAC* alteration is relatively limited.

This study has revealed a noticeable relationship between urban spatial patterns and runoff responses in small urbanized catchments. Our findings suggest that proper landscape planning strategy, such as impervious restriction, allocation optimization, and drainage optimization will benefit the task of urban flood management. 

## Figures and Tables

**Table 1:** Summary of spatial and drainage characteristics of the twelve study sites.

Catchment	Layout Type ^1^	Catchment Area (ha)	Impervious Fraction (%)		Percent Land Cover (%)				Average Drainage Area ^4^ (*A_d_*, ha)	Drainage Density ^5^ (m/ha)
			*TIA* ^2^	*DCIA* ^3^	Roof	Road	Tree	Lawn		
CAT 1	Linear	2.26	58.28	40.84	28.13	30.15	41.72	-	0.11	303.82
CAT 2	Interspersed	4.17	68.18	50.34	34.36	33.82	0.83	30.99	0.14	307.45
CAT 3	Semi-enclosed	4.81	73.68	65.11	33.78	39.89	22.03	4.29	0.11	316.86
CAT 4	Interspersed	1.39	54.38	24.06	30.32	24.06	45.62	-	0.09	303.08
CAT 5	Semi-enclosed	3.74	77.60	61.67	43.13	34.46	9.56	12.84	0.09	307.83
CAT 6	Interspersed	5.07	53.03	38.47	27.63	25.39	34.57	12.40	0.09	302.35
CAT 7	Linear	2.67	77.79	56.47	46.29	31.50	13.92	8.30	0.07	305.60
CAT 8	Linear	6.18	72.65	59.20	46.36	27.29	-	27.35	0.18	304.24
CAT 9	Interspersed	2.15	37.95	20.55	18.47	19.48	33.65	28.40	0.12	296.15
CAT 10	Semi-enclosed	6.84	74.50	71.42	38.59	35.91	2.31	23.20	0.16	305.39
CAT 11	Interspersed	2.73	48.56	19.27	34.94	13.62	12.17	39.27	0.16	302.13
CAT 12	Semi-enclosed	5.88	72.73	65.01	53.64	19.10	27.27	-	0.08	304.17

^1^ Three types of site layout were defined based on the categories of residential building form; ^2^ Total Impervious Area, *TIA*; ^3^ Directly Connected Impervious Area, *DCIA*; ^4^ Average drainage area (*A_d_*) expresses the average drainage area (ha) dominated by each rainwater inlet; ^5^ Drainage density expresses the total drainage pipe length (m) per unit drainage area (ha).

**Table 2:** Calibrated parameter values for the Storm Water Management Model (SWMM).

Land Cover	Manning’s Roughness	Depression Storage (mm)
Roads	0.017	0.675
Roofs	0.008	0.100
Lawns	0.266	1.540
Trees	0.150	1.540
Pipeline	0.0123	-

**Table 3:** Simulated total runoff depth (*Q_t_*, mm) and peak runoff depth (*Q_p_*, mm/min) of the twelve catchments.

Catchments	0.1 Year		0.15 Year		0.2 Year		0.3 Year		0.5 Year		1 Year		3 Year		5 Year		10 Year	
	*Q_t_*	*Q_p_*	*Q_t_*	*Q_p_*	*Q_t_*	*Q_p_*	*Q_t_*	*Q_p_*	*Q_t_*	*Q_p_*	*Q_t_*	*Q_p_*	*Q_t_*	*Q_p_*	*Q_t_*	*Q_p_*	*Q_t_*	*Q_p_*
CAT 1	3.29	0.08	4.29	0.15	9.15	0.27	14.19	0.40	21.97	0.66	32.50	1.02	49.52	1.68	57.48	2.01	68.53	2.46
CAT 2	4.22	0.13	5.45	0.23	11.66	0.40	16.79	0.57	24.23	0.87	34.78	1.25	51.82	1.90	59.97	2.22	71.01	2.66
CAT 3	7.46	0.16	9.54	0.30	18.79	0.51	25.36	0.74	33.67	1.09	45.10	1.54	62.98	2.26	71.50	2.60	82.72	3.08
CAT 4	1.84	0.05	2.43	0.10	7.72	0.22	12.99	0.42	20.86	0.83	31.40	1.32	48.58	2.12	56.67	2.50	67.71	3.02
CAT 5	4.87	0.10	6.21	0.18	13.20	0.33	19.22	0.49	27.31	0.77	38.28	1.14	55.68	1.79	63.98	2.11	74.96	2.55
CAT 6	3.04	0.08	3.91	0.15	8.39	0.25	12.54	0.36	19.48	0.55	29.41	0.82	45.99	1.33	53.89	1.58	64.75	1.94
CAT 7	4.79	0.10	6.28	0.17	13.95	0.33	19.85	0.52	27.82	0.85	38.88	1.25	56.45	1.92	64.68	2.25	75.90	2.70
CAT 8	4.71	0.11	5.97	0.20	12.08	0.34	17.31	0.49	24.59	0.72	35.10	1.03	52.08	1.56	60.17	1.82	71.17	2.19
CAT 9	1.59	0.04	2.09	0.08	5.35	0.14	8.70	0.22	15.17	0.40	24.75	0.66	40.99	1.12	48.85	1.36	59.55	1.69
CAT 10	5.68	0.12	7.17	0.22	14.04	0.38	19.31	0.56	26.92	0.83	37.60	1.19	54.86	1.81	63.05	2.12	74.17	2.54
CAT 11	1.76	0.04	2.75	0.09	7.41	0.22	11.96	0.36	19.33	0.64	29.60	0.99	46.59	1.60	54.29	1.90	65.30	2.33
CAT 12	5.23	0.16	6.63	0.28	13.38	0.45	18.70	0.64	26.34	0.95	37.05	1.35	54.39	2.04	62.54	2.38	73.59	2.85

**Table 4:** Regression models for total runoff depth (*Q_t_*, mm) and peak runoff depth (*Q_p_*, mm/min) with spatial pattern indicators.

Rainfall Condition	*Q_t_*		*Q_p_*	
	Regression Model	*R*^2^	Regression Model	*R*^2^
0.1 year	*Q_t_* = 0.900 × *DCIA* ** − 0.251	0.878	*Q_p_* = 0.002 × *DCIA* ** − 0.511 × *A_d_* ** + 0.005 *	0.926
0.15 year	*Q_t_* = 0.110 × *DCIA* **	0.857	*Q_p_* = 0.004 × *DCIA* ** − 0.918 × *A_d_* ** + 0.088 **	0.908
0.2 year	*Q_t_* = 0.181 × *DCIA* ** + 2.637	0.801	*Q_p_* = 0.006 × *DCIA* ** − 1.221 × *A_d_* * + 0.168 **	0.868
0.3 year	*Q_t_* = 0.309 × *TIA* ** − 3.383	0.808	*Q_p_* = 0.013 × *TIA* ** − 2.406 × *A_d_* ** − 0.043	0.915
0.5 year	*Q_t_* = 0.336 × *TIA* ** + 2.432	0.806	*Q_p_* = 0.016 × *TIA* ** − 3.674 × *A_d_* ** + 0.136	0.900
1 year	*Q_t_* = 0.368 × *TIA* ** + 10.969 *	0.804	*Q_p_* = 0.034 × *TIA* ** − 0.009 × *DCIA* * − 5.389 × *A_d_* ** + 0.048	0.919
3 year	*Q_t_* = 0.394 × *TIA* ** + 26.409 **	0.799	*Q_p_* = 0.050 × *TIA* ** − 0.017 × *DCIA* * − 7.567 × *A_d_* ** + 0.255	0.901
5 year	*Q_t_* = 0.407 × *TIA* ** + 33.645 **	0.799	*Q_p_* = 0.057 × *TIA* ** − 0.020 × *DCIA* * − 8.524 × *A_d_* ** + 0.391	0.892
10 year	*Q_t_* = 0.415 × *TIA* ** + 44.160 **	0.798	*Q_p_* = 0.065 × *TIA* ** − 0.024 × *DCIA* * − 9.719 × *A_d_* ** + 0.591	0.881

* Coefficient is significant at the 0.05 level; ** Coefficient is significant at the 0.01 level.

## References

[1] Palla A., Gnecco I. (2015). Hydrologic modeling of Low Impact Development systems at the urban catchment scale. J. Hydrol..

[2] Moglen G.E. (2009). Hydrology and impervious areas. J. Hydrol. Eng..

[3] Schueler T.R. (1994). The importance of imperviousness. Watershed Prot. Tech..

[4] Shuster W.D., Bonta J., Thurston H., Warnemuende E., Smith D.R. (2005). Impacts of impervious surface on watershed hydrology: A review. Urban Water J..

[5] Jha A.K., Bloch R., Lamond J. (2012). Cities and Flooding: A Guide to Integrated Urban Flood Risk Management for the 21st Century.

[6] Milly P., Wetherald R., Dunne K., Delworth T. (2002). Increasing risk of great floods in a changing climate. Nature.

[7] Zevenbergen C., Gersonius B. (2007). Challenges in urban flood management. Advances in Urban Flood Management.

[8] Meierdiercks K.L., Smith J.A., Baeck M.L., Miller A.J. (2010). Analyses of Urban Drainage Network Structure and its Impact on Hydrologic Response. J. Am. Water Resour. Assoc..

[9] Mejía A.I., Moglen G.E. (2009). Spatial patterns of urban development from optimization of flood peaks and imperviousness-based measures. J. Hydrol. Eng..

[10] Arnold C.L., Gibbons C.J. (1996). Impervious surface coverage: The emergence of a key environmental indicator. J. Am. Plan. Assoc..

[11] Schueler T.R., Fraley-McNeal L., Cappiella K. (2009). Is impervious cover still important? Review of recent research. J. Hydrol. Eng..

[12] Boyd M., Bufill M., Knee R. (1993). Pervious and impervious runoff in urban catchments. Hydrol. Sci. J..

[13] Dietz M.E., Clausen J.C. (2008). Stormwater runoff and export changes with development in a traditional and low impact subdivision. J. Environ. Manag..

[14] Yao L., Wei W., Chen L. (2016). How does imperviousness impact the urban rainfall-runoff process under various storm cases?. Ecol. Indic..

[15] Ogden F.L., Raj Pradhan N., Downer C.W., Zahner J.A. (2011). Relative importance of impervious area, drainage density, width function, and subsurface storm drainage on flood runoff from an urbanized catchment. Water Resour. Res..

[16] Bacchin T.K., Veerbeek W., Pathirana A., Denekew H.B., Zevenbergen C. (2011). Spatial metrics modeling to analyse correlations between urban form and surface water drainage performance. Proceedings of the 12nd International Conference on Urban Drainage.

[17] Qin H., Li Z., Fu G. (2013). The effects of low impact development on urban flooding under different rainfall characteristics. J. Environ. Manag..

[18] Li F., Duan H.F., Yan H., Tao T. (2015). Multi-Objective Optimal Design of Detention Tanks in the Urban Stormwater Drainage System: Framework Development and Case Study. Water Resour. Manag..

[19] Brander K.E., Owen K.E., Potter K.W. (2004). Modeled impacts of development type on runoff volume and infiltration performance. J. Am. Water Resour. Assoc..

[20] Hood M.J., Clausen J.C., Warner G.S. (2007). Comparison of Stormwater Lag Times for Low Impact and Traditional Residential Development. J. Am. Water Resour. Assoc..

[21] Yang G., Bowling L.C., Cherkauer K.A., Pijanowski B.C. (2011). The impact of urban development on hydrologic regime from catchment to basin scales. Landsc. Urban Plan..

[22] Van Nieuwenhuyse B.H.J., Antoine M., Wyseure G., Govers G. (2011). Pattern-process relationships in surface hydrology: Hydrological connectivity expressed in landscape metrics. Hydrol. Processes.

[23] Dams J., Dujardin J., Reggers R., Bashir I., Canters F., Batelaan O. (2013). Mapping impervious surface change from remote sensing for hydrological modeling. J. Hydrol..

[24] Faulkner D.S., Francis O., Lamb R. (2012). Greenfield run off and flood estimation on small catchments. J. Flood Risk Manag..

[25] Grimaldi S., Petroselli A., Romano N. (2013). Curve-Number/Green–Ampt mixed procedure for streamflow predictions in ungauged basins: Parameter sensitivity analysis. Hydrol. Processes.

[26] Grimaldi S., Petroselli A. (2014). Do we still need the Rational Formula? An alternative empirical procedure for peak discharge estimation in small and ungauged basins. Hydrol. Sci. J..

[27] Zoppou C. (2001). Review of urban storm water models. Environ. Model. Softw..

[28] Goldshleger N., Karnibad L., Shoshany M., Asaf L. (2012). Generalising urban runoff and street network density relationship: A hydrological and remote-sensing case study in Israel. Urban Water J..

[29] Lee J.G., Heaney J.P. (2003). Estimation of urban imperviousness and its impacts on storm water systems. J. Water Resour. Plan. Manag..

[30] Yao L., Chen L., Wei W. (2015). Assessing the effectiveness of imperviousness on stormwater runoff in micro urban catchments by model simulation. Hydrol. Processes.

[31] Fletcher T., Andrieu H., Hamel P. (2012). Understanding, management and modelling of urban hydrology and its consequences for receiving waters: A state of the art. Adv. Water Resour..

[32] Krebs G., Kokkonen T., Valtanen M., Setälä H., Koivusalo H. (2014). Spatial resolution considerations for urban hydrological modelling. J. Hydrol..

[33] Rossman L.A. (2010). Storm Water Management Model User's Manual, Version 5.0.

[34] Ouyang W., Guo B., Hao F., Huang H., Li J., Gong Y. (2012). Modeling urban storm rainfall runoff from diverse underlying surfaces and application for control design in Beijing. J. Environ. Manag..

[35] Vos P.E.J., Maiheu B., Vankerkom J., Janssen S. (2013). Improving local air quality in cities: To tree or not to tree?. Environ. Pollut..

[36] Wang Q., Zhang X., Wei M., Zhou Y., Li P., Bai G. (2011). Research summary of planning and design standards for storm water system in Beijing City. Water Wastewater Eng..

[37] Alley W.M., Veenhuis J.E. (1983). Effective impervious area in urban runoff modeling. J. Hydraul. Eng..

[38] Leopold L.B. (1968). Hydrology for Urban Land Planning: A Guidebook on the Hydrologic Effects of Urban Land Use.

[39] Boyd M.J., Bufill M.C., Knee R.M. (1994). Predicting pervious and impervious storm runoff from urban drainage basins. Hydrol. Sci. J..

[40] Sillanpää N., Koivusalo H. Impacts of urbanisation and event magnitude on runoff contributing area and runoff coefficients. Proceedings of the 13th International Conference on Urban Drainage.

[41] Guan M., Sillanpää N., Koivusalo H. (2015). Storm runoff response to rainfall pattern, magnitude and urbanization in a developing urban catchment. Hydrol. Processes.

[42] Sheeder S.A., Ross J.D., Carlson T.N. (2002). Dual urban and rural hydrograph signals in three small watersheds. J. Am. Water Resour. Assoc..

[43] Berne A., Delrieu G., Creutin J.-D., Obled C. (2004). Temporal and spatial resolution of rainfall measurements required for urban hydrology. J. Hydrol..

[44] Di Lazzaro M. (2009). Regional analysis of storm hydrographs in the Rescaled Width Function framework. J. Hydrol..

[45] Mejía A.I., Moglen G.E. (2010). Spatial distribution of imperviousness and the space-time variability of rainfall, runoff generation, and routing. Water Resour. Res..

[46] Jarden K.M., Jefferson A.J., Grieser J.M. (2015). Assessing the effects of catchment-scale urban green infrastructure retrofits on hydrograph characteristics. Hydrol. Processes.

[47] Stone B. (2004). Paving over paradise: How land use regulations promote residential imperviousness. Landsc. Urban Plan..

[48] Moussa R. (2008). What controls the width function shape, and can it be used for channel network comparison and regionalization?. Water Resour. Res..

[49] Gilroy K.L., McCuen R.H. (2009). Spatio-temporal effects of low impact development practices. J. Hydrol..

[50] Jia H., Yao H., Shaw L.Y. (2013). Advances in LID BMPs research and practice for urban runoff control in China. Front. Environ. Sci. Eng..

[51] Schmitt T.G., Thomas M., Ettrich N. (2004). Analysis and modeling of flooding in urban drainage systems. J. Hydrol..

[52] Carter T., Jackson C.R. (2007). Vegetated roofs for stormwater management at multiple spatial scales. Landsc. Urban Plan..

[53] Yao L., Chen L., Wei W., Sun R. (2015). Potential reduction in urban runoff by green spaces in Beijing: A scenario analysis. Urban For. Urban Green..

[54] Mejía A.I., Moglen G.E. (2010). Impact of the spatial distribution of imperviousness on the hydrologic response of an urbanizing basin. Hydrol. Processes.

[55] Aronica G., Lanza L. (2005). Drainage efficiency in urban areas: A case study. Hydrol. Processes.

[56] Walsh C.J., Roy A.H., Feminella J.W., Cottingham P.D., Groffman P.M., Morgan R.P. (2009). The urban stream syndrome: Current knowledge and the search for a cure. J. N. Am. Benthol. Soc..

[57] Nardi F., Annis A., Biscarini C. (2015). On the impact of urbanization on flood hydrology of small ungauged basins: The case study of the Tiber river tributary network within the city of Rome. J. Flood Risk Manag..

